# Structural and Physicochemical Characterization of Resistant Starch from Sixteen Banana Cultivars across Three Genome Groups

**DOI:** 10.3390/foods13203277

**Published:** 2024-10-16

**Authors:** Minhong Liang, Shiyun Tu, Jinfeng Fu, Juan Wang, Ou Sheng

**Affiliations:** 1 School of Food Science and Engineering, South China University of Technology, Guangzhou 510641, China; felmh@mail.scut.edu.cn (M.L.); sy.tu.scut@outlook.com (S.T.); jinfengfu0701@gmail.com (J.F.); 2 Key Laboratory of South Subtropical Fruit Biology and Genetic Resource Utilization, Ministry of Agriculture and Rural Affairs, Guangdong Provincial Key Laboratory of Science and Technology Research on Fruit Tree, Institute of Fruit Tree Research, Guangdong Academy of Agricultural Sciences, Guangzhou 510640, China

**Keywords:** banana resistant starch, genome group, crystalline pattern, thermal properties, pasting properties

## Abstract

Banana fruits are rich in starch, and unripe banana flour is considered a beneficial ingredient in the food industry because it has high levels of resistant starch, which significantly aids in promoting gut health and regulating blood sugar and lipid levels. However, the associations between banana cultivars with various genotypes cultivated globally and their resistant starch properties remain unclear. Herein, we investigated resistant starches from 16 banana cultivars covering three genome groups (ABB, AAB, and AAA) in order to reveal the differences and similarities among these cultivars. The results showed that there was a genotype-specific pattern in banana resistant starch (BRS) degradation. The AAA genome BRS exhibited a high degree of resistant starch degradation. The genotypes of the banana cultivars also impacted the granular morphology of the resistant starch. The ABB and AAB genome BRS were more conducive to forming resistant starch. The BRS samples from the three genome groups displayed either B-type or C-type structures. Even within the same genome group, the BRS samples exhibited differences in thermal and pasting properties. These findings reveal the impact of genotypes on BRS content and characteristics, providing a basis for future breeding and resistant starch utilization.

## 1. Introduction

The banana is a perennial herb that includes several species within the *Musa* genus under the Musaceae family. It is widely planted in both tropical and subtropical areas [1]. It has been estimated that nearly one-third of bananas are lost due to the public tendency to consume only ripened fruit [2]. Thus, using bananas at different ripening stages has gained interest over recent years. Unripe bananas contain a high level of starch, ranging from 61.00% to 77.12% in different cultivars, and in the pulp, the resistant starch accounts for more than 50% (dry base) [3,4]. Resistant starch has been reported to exhibit similar metabolic health benefits as dietary fiber, including significant roles in weight management, regulation of blood glucose and lipid levels, and the modulation of gut health [5,6]. Thus, the development of banana resistant starch (BRS) not only avoids a large waste of banana raw material but also has a large potential for commercial demand because of its dietary benefits.

The characteristics of banana starch are influenced by several factors, including cultivar, genome group, ripening stage, and environmental conditions [7,8,9,10]. There are about 300 kinds of banana cultivars worldwide and nearly 30 cultivars are grown in China, especially in Guangdong Province. Different cultivars may exhibit distinct genotypes, including the two naturally occurring diploid species (*M. acuminata* and *M. balbisiana*) and their hybrids [7]. Most of the banana cultivars that are widely grown today are triploids, including AAA, ABB, and AAB. Banana cultivars with varying genotypes can lead to significant differences in both the content of total starch and resistant starch during the postharvest ripening [11]. At early postharvest ripening, ABB genome bananas were reported to have higher levels of amylose and resistant starch [3]. Amylases, starch phosphorylase, and starch debranching enzyme were reported to be specifically up-regulated in AAB genome bananas, resulting in a faster starch degradation rate during ripening [12]. Moreover, the starch derived from banana cultivars has been analyzed for its structural characteristics, physicochemical properties, and potential applications [8]. For instance, Wang et al. [9] found significant differences in the pasting properties of banana starch among seven cultivars, while Marta et al. [10] demonstrated that pasting properties, color, and texture were strongly cultivar-dependent in banana starch from the AAA, AAB, and ABB genome groups. Hence, investigating the starch properties across various banana cultivars is crucial for understanding the specific starch characteristics associated with each genotype. This helps identify cultivars with a higher BRS content and better functional qualities, which can be used to develop new health-boosting products.

In this study, we selected 16 banana varieties from three different genome groups that are widely planted around the world. There are three cultivars from the ABB genome group, six from the AAB genome group, and seven from the AAA genome group. Previous research primarily focused on using banana flour to investigate the structural and physicochemical properties [3,7,8,9,10]. In contrast, this study directly extracted and analyzed the characteristics of resistant starch from the different banana cultivars. This approach allows for a more direct and targeted understanding of how various genotypes affect the characteristics of resistant starch. Changes in the starch content throughout the postharvest ripening were also assessed. This study could provide valuable insights for banana breeding and processing as well as the development of healthy food products based on BRS.

## 2. Materials and Methods

### 2.1. Banana Materials

Specific information about the banana samples used in this study is given in Table 1. Banana cultivars were supplied by the Institute of Fruit Tree Research, Guangdong Academy of Agricultural Sciences. Bananas from 16 cultivars harvested on the first day were collected and named as D1 samples. The remaining bananas from the same cultivars were treated with ethylene (100 μL/L) to facilitate ripening and collected on the seventh day, named as D7 samples.

### 2.2. Preparation of Banana Flour

The collected D1 and D7 banana samples were separated from the peel and sliced into pieces before being frozen in liquid nitrogen and stored at −80 °C for later use [1]. The frozen banana slices were dried at 60 °C for 72 h in a drying oven. Afterward, the slices were ground for 3 min using a milled machine and passed through a 100-mesh sieve to obtain banana flour. The resulting flour was placed in a desiccator to keep it dry. Then the contents of BRS, non-resistant starch (NRS), total starch, and predicted glycemic index (pGI) were determined for the D1 and D7 banana flours.

Amyloglucosidase-1, trypsin-α amylase, and D-glucose assay kit (GOPOD Format K-GLUK) were obtained from Shanghai Megazyme Bio-technology Co., Ltd. (Shanghai, China). Pectinase and amylase were supplied by Novozyme Biotechnology Co., Ltd. (Tianjin, China). Pepsin, porcine pancreatin, and amyloglucosidase-2 were available from Sigma-Aldrich Trading Co., Ltd. (Shanghai, China). All other reagents were analytical grade.

### 2.3. Isolation and Measurement of BRS

BRS samples were prepared using an enzymatic hydrolysis method according to Cheng Yanfeng et al. [13]. Banana flour samples (10 g) were weighed in centrifuge tubes with 100 mL of distilled water to obtain a slurry containing 10% (*m*/*m*) banana flour. The slurry was adjusted to pH 2.5 by 1 M HCl. Aliquots of 0.15% (*m*/*m*) Pectinase (Pectinex XXL, 10,000 PECTU/g) and 0.15% (*m*/*m*) amylase (300 AGU/mL) were added and incubated at 40 °C for 2 h to remove pectin, cellulose, protein, and digestible starch. The digested slurry was centrifuged at 3000 rpm for 15 min; the resulting precipitate was dried at 50 °C for 12 h and sieved through a 100-mesh screen. The sieved product was collected and stored in sealed containers at room temperature until needed. The D1 BRS samples were then used for analyzing their morphological and physicochemical properties, including polarizing microscopy, SEM, XRD, starch-iodine absorption spectra, thermal properties, and pasting properties.

BRS and NRS were analyzed using D-glucose assay kits under the AOAC Method [14]. Total starch content was the total of BRS and NRS. After adding trypsin-α amylase (3 Ceralpha Units/mg), banana flour (100 g) was kept shaking at 37 °C for 18 h, washed repeatedly with alcohol, and centrifuged; the sediment was transferred into a 100 mL volumetric flask and diluted to 100 mL with distilled water. Then the color of the obtained solution was developed with glucose chromogenic reagent (1.0 mg/mL) after adding amyloglucosidase-1 (3300 U/mL), and it was measured at 510 nm to obtain the absorbance in a UV-visible spectrophotometer (722S, Shanghai Jing Hua Technology Instruments Co., Ltd., Shanghai, China). Referring to the glucose standard curve, the glucose content was calculated and transformed to BRS, NRS, and total content.

### 2.4. In Vitro Digestion of Starch and Determination of pGI

A method by Woolnough et al. [15] with some modifications was used to determine the pGI value of banana flour samples. Banana flour (0.25 g) was placed into a 100 mL conical flask and 30 mL distilled water was inserted. The sample was incubated at 37 °C for 30 min with no stirring, and the pH adjusted to 2.5 by 1 M HCl if necessary. Then 0.8 mL of 2% pepsin (P7012, ≥2500 units/mg) was added and the sample was digested with slow constant stirring (130 rpm) to simulate gastric digestion conditions. After 30 min, 1 mL digestion solution and 4 mL absolute ethanol were pipetted into another 100 mL conical flask and mixed evenly. The pH of the mixed solution was adjusted to 6.2 with the addition of 1 mL 1 M NaHCO_3_, followed by 5 mL solution containing porcine pancreatin (8×USP specification) and amyloglucosidase-2 (A7095, ≥260 U/mL) (*v*:*v* = 1:1). The final volume was increased to 55 mL with distilled water. After that, aliquots of 1.0 mL were withdrawn at 0, 10, 20, 60, 120, and 180 min, added to centrifuge tubes containing 4 mL absolute ethanol, and centrifuged (3000 rpm, 10 min). The supernatant (0.1 mL) was taken to measure the amount of glucose by a glucose oxidase–peroxidase (GOPOD) kit. A standard reference was created by taking 0.25 g of white bread and following the same procedure as indicated above. The percentage of hydrolysis of the banana starch samples was calculated using Equation (1):

(1) $$\mathrm{Starch}\;\mathrm{hydrolysis}\;\left(\%\right)=\frac{\mathrm{At}\;\times 0.9\;}{B}\times 100\%$$

where At is the glucose content produced at t (h) and B is the total starch content. A conversion factor of 0.9 was used to convert molar mass from glucose to starch monomer units.

The starch hydrolysis curve was drawn with starch hydrolysis rate (%) as the ordinate and time (h) as the abscissa. In general, the curve followed the first-order equation and could be used to calculate the area under the hydrolysis curve (AUC). By dividing the AUC of starch samples by the AUC of a reference sample (white bread), the hydrolysis index (HI) of the samples was obtained. Then the predicted GI (pGI) was estimated using Equation (2) reported by Brennan C S et al. [16].

pGI = 0.862 HI + 8.192(2)

### 2.5. Normal Light and Polarizing Microscopy

BRS flour was dissolved in glycerol (50% concentration) and observed under a microscope (Vanox BHS-2, Olympus Corporation, Tokyo, Japan) using normal and polarized light.

### 2.6. Scanning Electron Microscopy (SEM)

The surface morphological properties of BRS particles were tested with a scanning electron microscope (Zeiss, Oberkochen, Germany) at an accelerating voltage of 2.0 kV and 500× magnification. BRS flour was fixed on an objective table coated with platinum (10–20 nm thickness).

### 2.7. X-ray Diffraction (XRD)

X-ray diffraction patterns were obtained with an X-ray diffractometer (D8 ADVANCE, Bruker, MA, USA) operating at 40 kV and 40 mA with Cu-Kα radiation (λ = 0.154 mm). The scanning region (2θ) was set from 4 to 60° with a step interval of 0.04° and a scan rate of 17.7 s per step. The method used in determining crystallinity was based on Cleven et al. [17] by calculating the ratio of the crystalline peak area to the total diffraction area.

### 2.8. Starch-Iodine Absorption Spectrum Determination

Spectra of iodine-bound starch samples were determined using the method given by Klucinec and Thompson [18]. The BRS (50 mg) and 10 mL 90% dimethyl sulfoxide (DMSO) solution were added to a 500 mL volumetric flask, followed by a water bath at 60 °C for 10 min, diluted with distilled water to 500 mL, and mixed. A 2 mL aliquot of the mixed solution, 25 mL distilled water, and 1 mL I_2_-KI (22 mg I_2_/mL, 44 mg KI/mL) were placed into a 50 mL volumetric flask and fixed to 50 mL with distilled water. Thereafter, the color of the obtained liquid was developed for 10 min. Scanning on each sample was performed from 500 to 800 nm with a UV-visible spectrophotometer (UV-1800, Shanghai Jing Hua Technology Instruments Co., Shanghai, China); λ_max_ for each sample was defined as the wavelength that resulted in the highest absorbance value.

### 2.9. Differential Scanning Calorimetry (DSC) Analysis

The thermal properties of BRS were tested using a differential scanning calorimeter (DSC 214 Polymer, NETZSCH, Bayern, Germany). BRS powders (10 ± 0.5 mg) were dispersed into 50 μL distilled water in an aluminum pan, hermetically sealed, and equilibrated for 24 h before the measurement. Thermal transitions of samples for gelatinization were marked by T_o_ (onset temperature), T_p_ (peak temperature), T_c_ (conclusion temperature), and ΔT (gelatinization temperature range).

### 2.10. Brabender Viscosity Measurement

A method developed by Aparicio-Saguilána, A et al. [19] was used to measure the viscosity curve of BRS using a Brabender viscometer (Visgraph-E, BRABENDER, Duisburg, Germany). BRS milk (BRS:distilled water = 6:100, *m*/*m*) was placed into a testing cup and stirred to homogenize the milk before running the test. The milk was heated from 30 °C to 95 °C at a rate of 7.5 °C/min and held for 5 min. Afterwards, it was cooled to 50 °C at a rate of 7.5 °C/min and kept at a constant temperature for 5 min. A continuous viscosity curve over time and temperature was automatically drawn.

### 2.11. Statistical Analysis

The data were analyzed using SPSS 21.0 (IBM), and the results were presented as the mean and standard deviation. One-way analysis of variance (ANOVA) was designed to compare the significant differences between the different banana samples. Clustering analysis and heat maps incorporating cluster analysis were produced using Origin 2024 (Origin Lab Company, Northampton, MA, USA) to classify the sixteen banana cultivars. The statistical significance was set at *p* < 0.05. Origin 2024 software was also used for graphics.

## 3. Results and Discussion

### 3.1. Banana Starch Content

During the postharvest ripening period, banana flour samples from 16 cultivars across the three genome groups (ABB, AAB, and AAA) exhibited significant differences in BRS, NRS, and total starch contents between D1 and D7 (*p* < 0.05) (Table 2). The BRS content of the 16 cultivars decreased gradually, ranging from 42.39 to 96.74 g/100 g, while the NRS content showed an increased range of 2.03–27.63 g/100 g. A decline in total starch content was also observed across the 16 banana cultivars during the ripening. These results show that the unripe banana cultivars have a high BRS content and total starch content at an early postharvest ripening period, which was then converted into soluble sugar content through an enzymatic breakdown during the postharvest ripening [20]. The BRS of Meishi 2 (AAB), Jinfen 1 (ABB), and Maiden (AAB) cultivars (96.74, 90.92, and 90.10 g/100 g, respectively) at D1 were significantly higher (*p* < 0.05) than those of the remaining 13 cultivars. In contrast, unripe mango, another climacteric fruit, had a lower resistant starch content of only 68.6% [21]. It was noteworthy that during the postharvest ripening, the mean decrease in the proportion of BRS in total starch was 56.06% in the ABB genome group and 55.62% in the AAB genome group, while in AAA genome bananas, the proportion of BRS in total starch was reduced by an average of 77.66%. The ratio of BRS in total starch reduced more slowly in ABB and AAB genome bananas than in AAA genome bananas, likely due to differences in the internal starch structure and the activity of amylases [22,23]. A genotype-specific pattern in resistant starch degradation was observed among the 16 banana cultivars. Compared to AAB genome and ABB genome BRS, AAA genome BRS was more susceptible to enzyme digestion, resulting in degradation widely.

**Figure 1:**
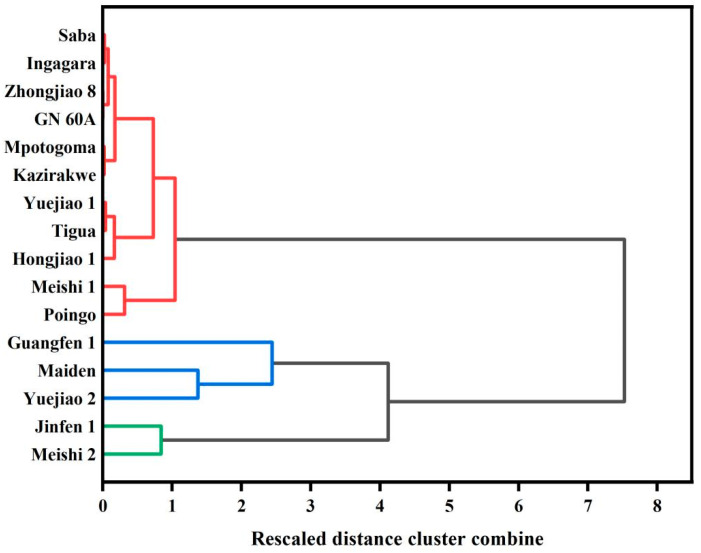
Results of hierarchical cluster analysis of BRS from 16 banana cultivars at D1 and D7. The lines of the same color in the figure represent that these banana cultivars are classified into the same cluster.

The hierarchical cluster analysis based on the content of BRS at D1 and D7 was carried out to explore the differences and similarities among the banana cultivars with different genotypes (i.e., ABB, AAB, AAA) (Figure 1, Table 3). The 16 banana cultivars were divided into cluster 1 (C1), cluster 2 (C2) and cluster 3 (C3). C1 contained two banana cultivars including Meishi 2 (AAB) and Jinfen 1 (ABB) with a high BRS content at both D1 and D7. C2 was further divided into two subgroups, C2A and C2B. C2A contained Yuejiao 2 (AAA) and Maiden (AAB) with a moderately high BRS content at D1 and a moderately low BRS content at D7. Guangfen 1 (ABB) was divided into C2B with a moderately high BRS content at D1 and a high BRS content at D7. C3 had 11 banana cultivars with a low BRS content, from 48.79 g/100 g to 65.93 g/100 g, at D1 and almost no BRS content, from 0 g/100 g to 12.4 g/100 g, at D7; it then was divided into three subgroups according to the different BRS content at D1 and D7. We could find that cultivars with the same genotype, such as Jinfen 1 and Guangfen 1, were distributed into different groups, indicating that cultivars of the same genotype had different BRS contents. In addition, the majority of the AAA genome bananas, including Kazirakwe, Mpotogoma, Ingagara, GN 60A, and Zhongjiao 8, presented moderate to low BRS content. The results pointed out that though the impact of distinct genotypes on the starch content at D1 of the banana cultivars remains uncertain, it is possible that the AAA genotype promoted BRS degradation during the postharvest ripening.

### 3.2. Comparison of the Predicted Glycemic Index (pGI) of BRS

The glycemic index (GI) is an index that reflects the degree and variation of the impact of intake food on human blood sugar. The lower the GI value, the lower the blood sugar intake. Since measuring the GI value must be performed in vivo, it shows great fluctuation and is time-consuming and costly; as a result, current studies mostly replace the GI value with the pGI value [15,24]. Using an in vitro digestion method, the pGI test simulates the digestive environment and measures the release of glucose from food to estimate the GI [25]. According to the GI value, the pGI value of food can be divided into three levels: low GI (GI < 55), medium GI (55–70), and high GI (GI > 70) [26].

The pGI values measured from the 16 BRS samples at D1 varied between 9.71–16.47 and were far less than 55, so they belonged to the low GI value range (Table 2). In addition, a significant correlation (*p* < 0.05) between the pGI values and banana cultivars could be observed in the present work. Kzairakwe, Jinfen 1, and Saba recorded higher pGI values, while Meishi 2 exhibited the lowest. The pGI was reported to correlate with the presence of resistant starch. As shown by Agama-Acevedo et al. [27], adding unripe banana flour to biscuits resulted in a decrease in the GI of biscuits, since the resistant starch contributed to slowing down the rate of enzymatic hydrolysis of starch. In addition, during baking, the structure of the natural starch particles tended to be disturbed and were easily hydrolyzed by digestive enzymes. However, adding BRS reduced the proportion of fast digestible starch and inhibited the hydrolysis process [28]. It showed that postprandial hyperglycemia and high insulin spikes caused by the rapid digestion of starch were suppressed by the resistant starch [29]. The lower the GI values of the resistant starch, the better the stability of the postprandial blood glucose. Therefore, unripe BRS could be a great potential dietary source for people with hyperglycemia, consistent with the previous research [4,7]. Meishi 2 emerged as the most suitable BRS cultivar with the lowest pGI. Furthermore, the pGI of AAB genome BRS was moderately low, with an average of 11.68. In comparison, the mean pGI values of ABB and AAA genome BRS were 13.91 and 13.15, respectively.

### 3.3. Granule Morphology

**Figure 2:**
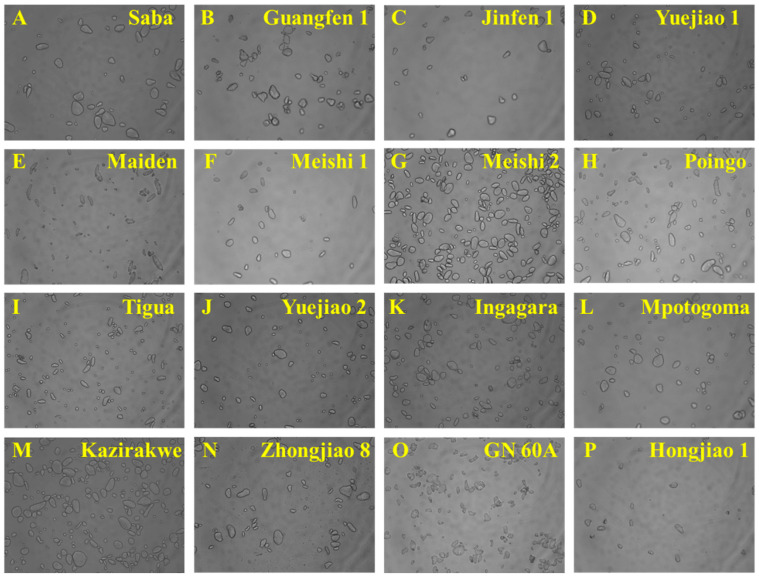
Optical microscopy images (×200) of BRS from 16 banana cultivars.

**Figure 3:**
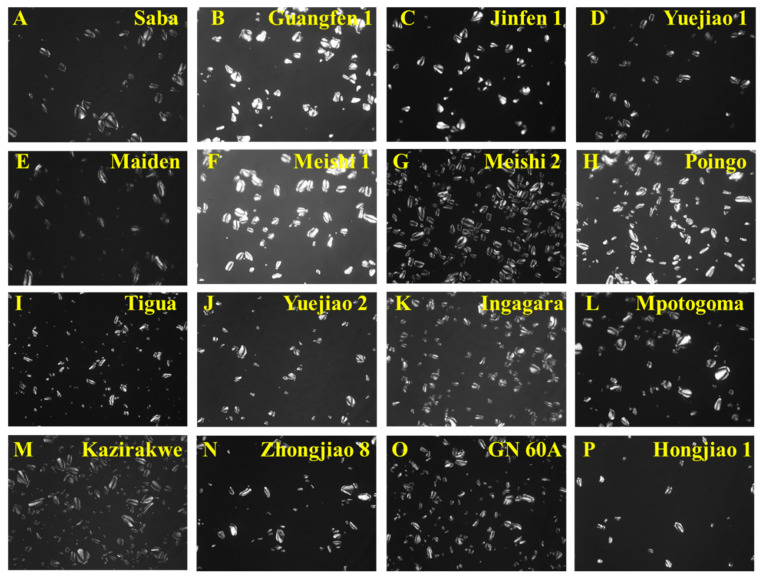
Polarized light microscopy images (×200) of BRS from 16 banana cultivars.

**Figure 4:**
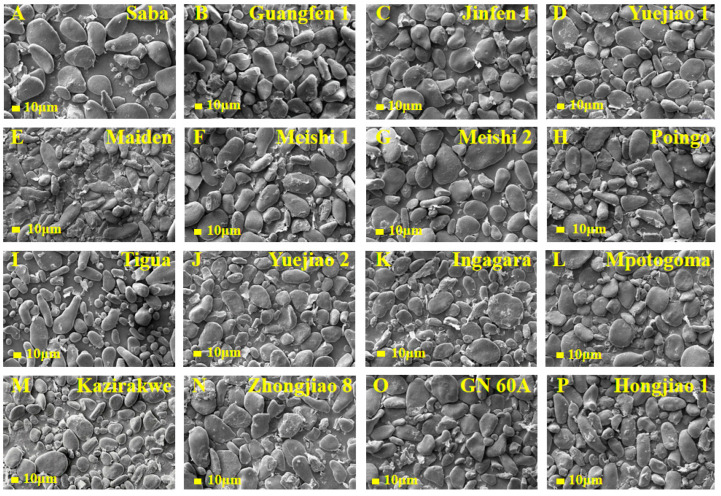
Scanning electron microscopy images (×500) of BRS from 16 banana cultivars.

The morphology of 16 BRS sample particles was observed to have ring patterns with different particle shapes, recorded as flat, irregular oval, slender rod, and cone shaped (Figure 2), consistent with the findings from a previous study [30]. All the particles had birefringence without distinct differences under the polarized light, appearing as Maltese crosses (Figure 3). The Maltese crosses were attributed to the existence of the radial alignment of the crystalline structures in the BRS particles, suggesting that intact crystalline structures were retained during the starch extraction. Through SEM analysis, more details of the starch granules, including size and shape, the presence of other compounds, structural integrity, and surface morphology, were analyzed (Figure 4). The BRS granules of the 16 banana cultivars from the three genome groups displayed a wide range of particle sizes (10 μm–60 μm) and a tight arrangement. Parts of the BRS granules had smooth and unbroken surfaces, while there were also some having cracks and wrinkles on the surface with sheet fragments attached. This phenomenon may be explained by the fact that during the early postharvest ripening, cell walls consisting of pectin, cellulose, and a small amount of protein were left over the surface after starch degradation [31].

Four groups were created from the 16 BRS samples according to their microstructure at D1. Maiden (AAB), Poingo (AAB), Tigua (AAB), and Hongjiao 1 (AAA) made up the first group with numerous elongated granules. Tigua and Poingo exhibited identical genotypes (AAB), but the presence of a greater number of small particles in Tigua made it more susceptible to enzymatic digestion [32], resulting in wider BRS degradation for Tigua than for Poingo. The particles in Maiden were of a small size and densely arranged with a low degradation. This suggests that the particle sizes and arrangement have an impact on the rate of starch digestion. The second group was distinguished by an oval shape, including Yuejiao 1 (AAB), Meishi1 (AAB), Meishi 2 (AAB), Yuejiao 2 (AAA), Ingagara (AAA), Saba (ABB), and Mpotogoma (AAA) cultivars. The large round starch granules of the AAB genome BRS (except Yuejiao 1) tended to have a low starch degradation. The cultivars with a high percentage of irregular shapes were sorted out and classified into the third group, including Jinfen 1 (ABB), Guangfen 1 (ABB), Kazirakwe (AAA), Zhongjiao 8 (AAA), and GN 60A (AAA) cultivars. The AAA genome BRS (Kazirakwe, Zhongjiao 8, and GN 60A) tended to contain a greater number of small granules, which aligned with the result of high degradation from the BRS analysis (Table 2). These results indicate that the variations in granular morphology might be accredited to the genotypes of the cultivars. Furthermore, the differences in the BRS microstructures among the 16 banana cultivars were likely to affect the physicochemical properties.

### 3.4. Crystalline Structure

**Figure 5:**
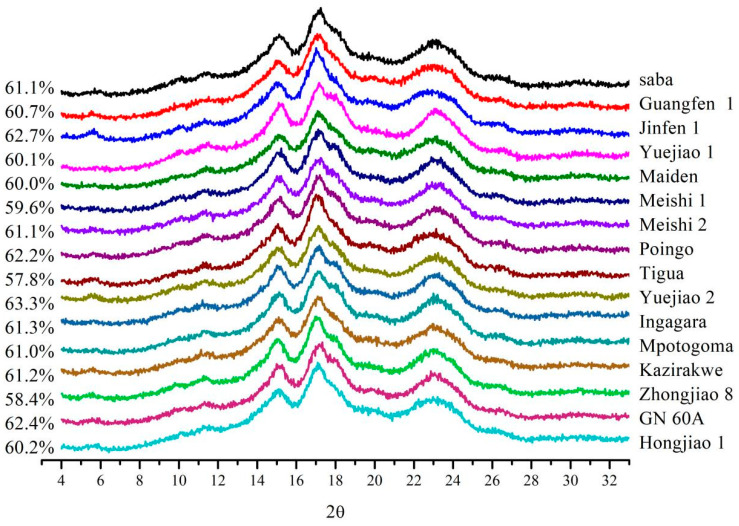
X-ray diffraction patterns and crystallinity indexes of BRS from 16 banana cultivars.

X-ray diffraction patterns and crystalline parameters of the BRS from the 16 banana cultivars covering different genome groups are presented in Figure 5. These patterns were used to study the type and level of BRS crystallinity over the different cultivars. Previous studies have reported that natural banana starch exhibits three types of crystal structures, including type A [33], type B [10,34], and type C [35]. In our study, all particles were found to exhibit a weak diffraction peak at 5.6 °θ and a broad peak ranging from 22° to 24 °θ, suggesting that they were B- or C-type crystals rather than A-type crystals. Saba (ABB), Jinfen 1 (ABB), Meishi 2 (AAB), Poingo (AAB), and GN 60A (AAA) were identified as C-type crystals with a weak peak at 5.7°, moderate peaks at 15.0°, 19.5°, and 22.0 °θ, and a strong peak at 17.0 °θ. The C-type pattern was a mixture of A-type crystal and B-type crystal. The other 11 BRS samples contained reflections at 2θ values of 5.6°, 15.0°, 17.0°, and 23.0 °θ, which are attributed to the B-type crystal. These results revealed that the different BRS granules had different crystal types. Even samples of BRS with the same genome had different crystals, showing the banana genotypes did not significantly impact the crystalline pattern of the starch. Instead, the growth conditions of the banana cultivars were considered to play a crucial role in shaping the crystalline structure of the starch [10].

To estimate the degree of structural order, the crystallinity index (CI) of the BRS granules from the 16 banana cultivars was calculated. The BRS granules ranged between 57.8~63.3%, which were much higher than the cultivars of India (2.04–12.22%) reported by [3] and Thailand (24.2–31.3%) reported by Vatanasuchart et al. [36]. It was reported that the double-helical arrangement of the branched-chain structures in the crystalline region determined the CI value, relevant to enzyme hydrolysis [37]. In our study, the CI values of the BRS from the 16 banana cultivars were close to the same, with a ratio of maximum to minimum of 1.09. In addition, Zhou et al. [38] found that the B-type and C-type crystals of the resistant starch were more compact and resistant to enzymatic digestion. The BRS samples from the three genome groups exhibited B-type or C-type structures, suggesting they were less affected by digestive enzymes in the glycemic index experiment, which might explain the low pGI values (Table 2).

### 3.5. Starch-Iodine Absorption Spectra

**Figure 6:**
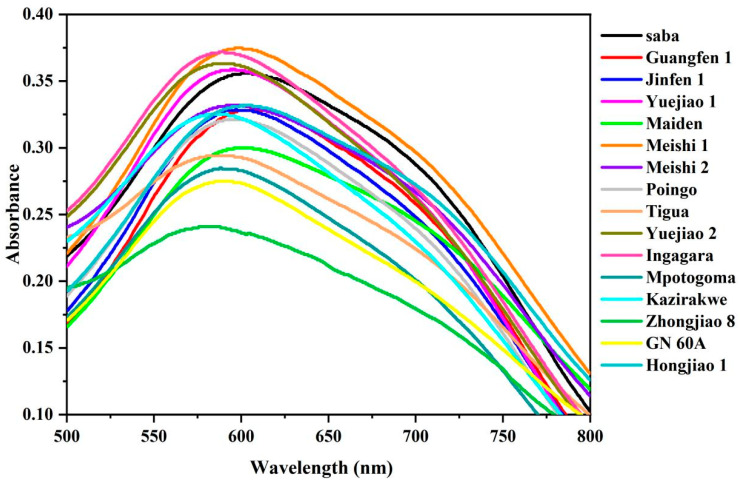
Iodine absorption spectra from BRS of 16 BRS cultivars.

The absorption of the starch samples and iodine was attributed to the content of amylose and amylopectin. The combination of amylose and iodine produced a spiral complex which has a maximum absorption peak in the wavelength of 620–680 nm, while amylopectin formed a complex with iodine, which has a maximum absorption peak of 520–560 nm [39]. In our study, the iodine absorption spectra of the 16 BRS samples from the ABB, AAB, and AAA genome groups were obtained and are shown in Figure 6. The maximum absorption wavelengths of the BRS samples varied from 582 nm to 606 nm, which was between the maximum absorption peaks of amylose and amylopectin. The results indicated that all samples comprised both amylose and amylopectin. The value of the optical density (OD) at 620 nm (OD620) is usually used to assess the amylose content of starch [40]. It was anticipated that Meishi 1 (AAB) would exhibit a high amylose content, as indicated by the highest OD620 value. Conversely, Zhongjiao 8 (AAA) was expected to have the lowest amylose content, as indicated by the lowest OD620 value. Moreover, the ratio of amylose to amylopectin, as well as the weight of amylose molecules, impacts the aggregation of amylose molecules, resulting in different absorption peaks [41,42]. Guangfen 1 (ABB) had the largest absorption wavelength (606 nm), followed by Saba (ABB), Maiden (AAB), Hongjiao 1 (AAA), Meishi 1 (AAB), Meishi 2 (AAB), and Poingo (AAB), among which Zhongjiao 8 (AAA) reached the lowest value (582 nm). The results indicated that the BRS samples from the ABB and AAB genome groups exhibited a greater inclination to form resistant starch than those from the AAA genome group.

### 3.6. Thermal Properties

A thermal analysis technique was employed to examine the starch structural changes that occur during the thermal transformation of distinct starch particles. The thermal parameters of the 16 BRS samples re summarized in Table 4. The BRS samples from the ABB, AAB, and AAA genome groups had different gelatinization temperatures. The gelatinization temperatures of T_o_, T_p_, and T_c_ ranged from 48.1 to 68.6 °C, from 71.7 to 82.4 °C, and from 76.7 to 87.0 °C among the 16 banana cultivars, respectively. The gelatinization ∆T varied from 11.9 to 33.6 °C. Different T_o_ values were observed in the same genome group. Kazirakwe (AAA), with a low OD620 value, exhibited the highest gelatinization T_o_, whereas Yuejiao 2 (AAA), with a high OD620 value with more amylose, had the lowest gelatinization T_o_. This phenomenon could be ascribed to the BRS samples from the same genome group varying in amylose content. The lower amylose content granules displayed a higher relative crystallinity, causing a higher temperature to disrupt them [43]. All the BRS samples of the 16 banana cultivars had a T_p_ greater than 70 °C. Though both GN 60A (AAA) and Saba (ABB) had the same crystallinity for B-type, GN 60A had the largest T_p_, while Saba was noted to be the lowest. As shown in Table 4, we found that Tigua (AAB), with the lowest T_o_ (high amylose), had the highest ∆T value, marking a more stable crystal pattern during the gelatinization. The ∆T value reflected the uniformity and stability of the BRS. According to Chávez-Salazar et al. [44], wide gelatinization ranges are mainly due to a higher heterogeneity and the differences between the arrangement of starch components in the BRS particles. Therefore, the thermal properties of the BRS were speculated to connect with the amylose content, genome group, and growing conditions.

### 3.7. Pasting Properties

The pasting property parameters, including pasting viscosity and temperature, exhibited differences among the 16 BRS flours from the three genome groups (Table 5). As the temperature rose, the granules underwent swelling and reached a point where they collided with one another, coupled with an increase in the viscosity of the samples. A similar initial gelatinization temperature (A) was observed among the 16 BRS samples, ranging between 72.7–79.4 °C. Meishi 2 (AAB) had a high A (75.7 °C), needing a higher temperature for pasting. The process of increasing viscosity continued until the peak viscosity (B) was reached, which reflected the swelling of the starch particles, and the higher peak viscosity also indicated that the starch paste had a greater viscosity [45]. The starch granules swelled to the maximum volume and then ruptured, resulting in a moderate decline until the viscosity reached its lowest point (D), which reflected the ability to resist shear-rupturing. Following the decline in temperature, the water molecules surrounded by amylose and amylopectin became less robust, resulting in an increase in viscosity. The final viscosity (F) reflected the hardness of the starch glue at room temperature. During gelatinization, Tigua (AAB) had the highest B (328 BU), C (315 BU), D (267 BU), E (360 BU), and F (358 BU), while Maiden (AAB) had the lowest B (62 BU), C (60 BU), D (57 BU), E (68 BU), and F (71 BU). It was postulated that the same genome BRS samples exhibited a variation in starch components, which led to different thermal and pasting properties. The breakdown (B-D) reflected the stability of the starch particles during heating. The starch structures tended to be unstable at a higher breakdown [46], since granule structures became loose during the pasting process. The lowest B-D was in Maiden (AAB), which was inferred to exhibit the most stability among all the samples. In addition, there was a correlation between the crystalline type and the thermal stability of the starch. Generally, A-type crystallization was the best, followed by C-type and B-type [47]. In the present study, Meishi 2 (AAB), belonging to the C-type crystal group, had the highest B-D, whereas Maiden (AAB), with a B-type crystal, seemed to be more stable. It was supposed that crystallization might not be the decisive factor for the thermal stability of the BRS samples. The setback value (E-D) was used to judge the texture and digestibility of the starch [46]. Tigua (AAB) showed a pronounced setback (retrogradation), with the highest value (93) during cooling, suitable for instant noodle and rice noodle production.

### 3.8. Clustered Heat Map Analysis on BRS Characteristics

**Figure 7:**
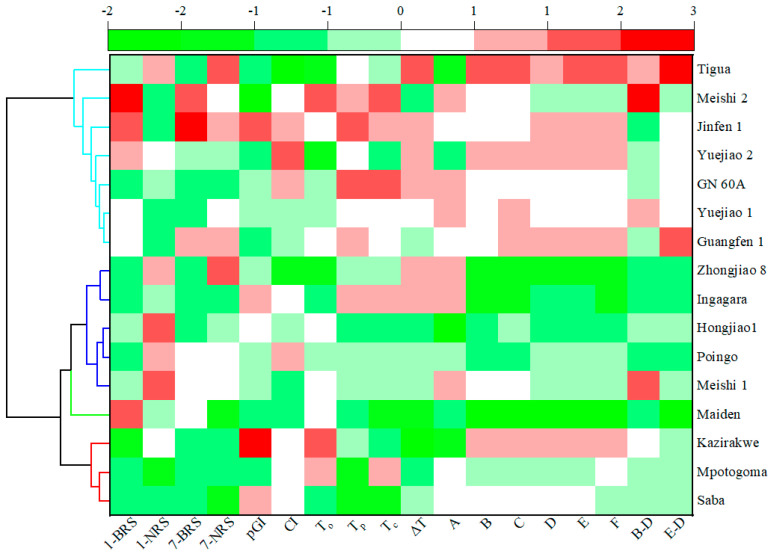
Heat map with dendrogram of BRS from 16 banana cultivars. The lines of the same color in the figure represent that these banana cultivars are classified into the same cluster. 1-BRS, resistant starch content of D1 banana flour; 1-NRS, non-resistant starch content of D1 banana flour; 7-BRS, resistant starch content of D7 banana flour; 7-NRS, non-resistant starch content of D7 banana flour; pGI, predicted glycemic index of banana flour; CI, crystallinity of resistant banana starch; T_o_, onset temperature; T_p_, peak temperature; T_c_, conclusion temperature; ΔT, the gelatinization temperature range; A, initial gelatinization temperature; B, peak viscosity (BU); C, viscosity at 95 °C (BU); D, viscosity after 5 min at 95 °C (BU); E, viscosity at 50 °C (BU); F, viscosity after 5 min at 50 °C (BU); B-D: breakdown viscosity; E-D: retrogradation viscosity.

Heat maps incorporating cluster analysis were produced using Origin 2024. Before the analysis of the data, all the data were normalized for z-values because of the differences in units from data to data. For the hierarchical cluster analysis, Ward’s method and squared Euclidean distance were chosen as the clustering method and similarity measure, respectively. The data, including RS content, NRS content, pGI, CI, T_o_, T_p_, T_c_, ΔT, A, B, C, D, E, F, B-D, and E-D values, were finally classified for the 16 banana cultivars from the ABB, AAB, and AAA genome groups into four clusters (Figure 7, Table 6). Saba (ABB), Mpotogoma (AAA), and Kazirakwe (AAA) were divided into cluster 1 (C1), with less BRS and NRS in both D1 and D7, indicating that the cultivars in C1 had low starch degradation. Maiden (AAB) was divided into cluster 2 (C2) and exhibited a high BRS in D1, while all other characteristic indicators were low. Cluster 3 (C3) included Meishi 1 (AAB), Poinga (AAA), Hongjiao 1 (AAA), Ingagara (AAA), and Zhongjiao 8 (AAA), with low pasting and thermal parameters. Other cultivars (Guangfen 1 (ABB), Yuejiao 1 (AAB), GN 60A (AAA), Yuejiao 2 (AAA), Jinfen 1 (ABB), Meishi 2 (AAB), and Tigua (AAB)), with high pasting and thermal parameters, were divided into cluster 4 (C4) and were less susceptible to destruction by high temperatures. It is noteworthy that some cultivars with the same genotype, such as Saba, Jinfen 1, and Guangfen 1, having ABB genome BRS, were distributed into different clusters, indicating that cultivars from different genotypes had different starch properties.

## 4. Conclusions

During the postharvest ripening period, the BRS from the ABB and AAB genome groups were more resistant to enzymatic digestion. In addition, they had a higher amylose-to-amylopectin ratio and were more likely to form resistant starch. In contrast, the BRS from the AAA genome group had a high degradation and tended to contain a greater number of small granules. These findings contribute to identifying BRS with a high content and a stable structure, providing important insights for banana breeding and the development of BRS health-boosting products. Regrettably, this study did not include the separation and measurement of amylose and amylopectin in the banana samples; further research could focus on these components to clarify their relationship with genotypes and physicochemical characterization.

## Figures and Tables

**Table 1:** Details of banana samples used in this study.

Cultivar Name	Cultivated Group	Sub-Type	Genome
Saba	Saba	Saba	ABB
Guangfen 1	Pisang Awak	Pisang Awak	ABB
Jinfen 1	Pisang Awak	Pisang Awak	ABB
Yuejiao 1	Plantain	French	AAB
Maiden	Plantain	French	AAB
Meishi 1	Plantain	French Horn	AAB
Meishi 2	Plantain	French Horn	AAB
Poingo	Maoli-Popoulu	Maoli-Popoulu	AAB
Tigua	Iholena	Iholena	AAB
Yuejiao 2	EAHB	Mbidde	AAA
Ingagara	EAHB	Nakitembe	AAA
Mpotogoma	EAHB	Musakala	AAA
Kazirakwe	EAHB	Nakabululu	AAA
Zhongjiao 8	Cavendish	Giant Cavendish	AAA
GN 60A	Cavendish	Dwarf Cavendish	AAA
Hongjiao 1	Red and Green	Red	AAA

**Table 2:** Content of BRS, NRS, and total starch in banana flour at D1 and D7, and the value of pGI at D1, of 16 banana cultivars.

Sample	BRS (g/100 g)		NRS (g/100 g)		Total Starch (g/100 g)		BRS/Total Starch (%)		pGI
	D1	D7	D1	D7	D1	D7	D1	D7	D1
Saba	48.79 ± 5.70 ^fg^	1.43 ± 1.56 ^e^	5.76 ± 0.15 ^fg^	7.88 ± 1.25 ^j^	54.50 ± 5.81 ^fg^	9.28 ± 1.74 ^h^	89.3 ± 2.54 ^bd^	13.19 ± 17.71 ^ef^	14.68 ± 0.27 ^c^
Guangfen 1	68.69 ± 4.30 ^cd^	23.92 ± 0.89 ^b^	6.41 ± 3.47 ^fg^	34.05 ± 0.21 ^bc^	75.09 ± 4.72 ^ce^	57.96 ± 1.10 ^b^	91.47 ± 1.91 ^ac^	41.24 ± 0.95 ^bc^	11.72 ± 0.23 ^hi^
Jinfen 1	90.92 ± 3.19 ^ab^	38.91 ± 2.97 ^a^	6.21 ± 0.80 ^fg^	36.41 ± 2.13 ^ab^	97.25 ± 3.43 ^ab^	75.32 ± 3.03 ^a^	93.48 ± 0.65 ^ab^	51.62 ± 3.35 ^ab^	15.32 ± 0.26 ^b^
Yuejiao 1	65.93 ± 5.90 ^ce^	0.00 ± 0.00 ^e^	4.46 ± 0.33 ^g^	31.56 ± 2.27 ^cd^	70.52 ± 7.71 ^cf^	31.56 ± 2.27 ^d^	93.50 ± 1.29 ^ab^	0.00 ± 0.00 ^f^	12.19 ± 0.13 ^gh^
Maiden	90.10 ± 8.03 ^ab^	15.24 ± 3.73 ^c^	8.90 ± 0.47 ^ef^	11.25 ± 2.57 ^ij^	98.94 ± 9.03 ^a^	26.49 ± 2.51 ^ef^	91.02 ± 1.07 ^ac^	57.10 ± 12.42 ^a^	11.44 ± 0.35 ^i^
Meishi 1	59.09 ± 2.80 ^dg^	9.39 ± 4.43 ^cd^	27.63 ± 0.26 ^a^	28.76 ± 0.88 ^de^	86.73 ± 3.50 ^ac^	38.16 ± 3.75 ^c^	68.08 ± 1.38 ^g^	23.78 ± 10.74 ^de^	12.40 ± 0.09 ^fg^
Meishi 2	96.74 ± 3.43 ^a^	28.42 ± 5.67 ^b^	3.64 ± 0.40 ^g^	31.75 ± 2.89 ^cd^	100.38 ± 3.15 ^a^	60.17 ± 2.78 ^b^	96.35 ± 1.47 ^a^	46.90 ± 8.62 ^ab^	9.71 ± 0.30 ^j^
Poingo	50.33 ± 15.62 ^eg^	12.40 ± 3.00 ^c^	19.35 ± 0.20 ^bc^	26.94 ± 0.93 ^ef^	69.69 ± 17.07 ^df^	39.34 ± 2.17 ^c^	70.16 ± 10.33 ^g^	31.19 ± 7.49 ^cd^	12.72 ± 0.27 ^ef^
Tigua	62.50 ± 2.27 ^cf^	0.00 ± 0.00 ^e^	22.41 ± 2.51 ^b^	40.16 ± 2.61 ^a^	84.92 ± 2.43 ^ad^	40.16 ± 2.61 ^c^	73.58 ± 1.18 ^fg^	0.00 ± 0.00 ^f^	11.61 ± 0.14 ^i^
Yuejiao 2	77.35 ± 2.44 ^bc^	4.01 ± 0.35 ^de^	15.98 ± 3.40 ^cd^	25.71 ± 0.69 ^ef^	93.33 ± 2.00 ^ab^	29.73 ± 0.93 ^de^	82.86 ± 1.37 ^de^	13.50 ± 1.05 ^ef^	11.60 ± 0.23 ^i^
Ingagara	47.22 ± 3.49 ^fg^	0.00 ± 0.00 ^e^	13.13 ± 2.37 ^de^	17.87 ± 0.23 ^g^	60.34 ± 3.57 ^eg^	17.87 ± 0.23 ^g^	78.17 ± 1.85 ^ef^	0.00 ± 0.00 ^f^	14.15 ± 0.08 ^d^
Mpotogoma	44.72 ± 4.05 ^g^	0.00 ± 0.00 ^e^	2.03 ± 2.13 ^g^	16.81 ± 0.45 ^gh^	46.75 ± 4.31 ^g^	16.81 ± 0.45 ^g^	95.58 ± 1.08 ^ab^	0.00 ± 0.00 ^f^	11.58 ± 0.25 ^i^
Kazirakwe	42.39 ± 8.98 ^g^	0.00 ± 0.00 ^e^	14.55 ± 0.99 ^d^	18.17 ± 0.61 ^g^	56.87 ± 9.31 ^fg^	18.17 ± 0.61 ^g^	73.67 ± 6.54 ^fg^	0.00 ± 0.00 ^f^	16.47 ± 0.06 ^a^
Zhongjiao 8	52.43 ± 4.20 ^dg^	0.00 ± 0.00 ^e^	21.13 ± 0.92 ^b^	39.25 ± 0.55 ^a^	73.73 ± 3.76 ^ce^	39.25 ± 0.55 ^c^	70.99 ± 3.05 ^g^	0.00 ± 0.00 ^f^	12.58 ± 0.06 ^fg^
GN 60A	52.52 ± 3.61 ^dg^	0.00 ± 0.00 ^e^	9.41 ± 2.14 ^ef^	14.03 ± 0.64 ^hi^	61.93 ± 4.26 ^eg^	14.03 ± 0.64 ^gh^	84.81 ± 1.23 ^ce^	0.00 ± 0.00 ^f^	12.45 ± 0.08 ^fg^
Hongjiao 1	57.37 ± 4.98 ^dg^	0.00 ± 0.00 ^e^	23.23 ± 1.20 ^ab^	23.64 ± 0.14 ^f^	80.61 ± 5.90 ^bd^	23.64 ± 0.14 ^f^	71.06 ± 1.88 ^g^	0.00 ± 0.00 ^f^	13.20 ± 0.12 ^e^

Data expressed as the mean and standard deviation. Different superscript letters in a column indicate significant differences (*p* < 0.05, *n* = 3). BRS, resistant starch; NRS, non-resistant starch; pGI, predicted glycemic index.

**Table 3:** Results of hierarchical cluster analysis of BRS from 16 banana cultivars at D1 and D7.

Cluster	Cultivar	Genotype
C1	Meishi 2, Jinfen 1	AAB, ABB
C2	A-Maiden, Yuejiao 2	AAB, AAA
	B-Guangfen 1	ABB
C3	A-Meishi 1, Poingo	AAB, AAB
	B-Hongjiao 1, Tigua, Yuejiao 1	AAA, AAB, AAB
	C-Kazirakwe, Mpotogoma, Ingagara, Saba, GN 60A, Zhongjiao 8	AAA, AAA, AAA, ABB, AAA, AAA

**Table 4:** Differential scanning calorimetry analysis of BRS from 16 banana cultivars.

Sample	T_o_ (°C)	T_p_ (°C)	T_c_ (°C)	ΔT (°C) (Tc − T_o_)
Saba	54.5 ± 16.1	71.7 ± 13.4	76.7 ± 11.5	22.2 ± 16.2
Guangfen 1	61.8 ± 9.7	79.8 ± 2.1	84.2 ± 2.1	22.4 ± 8.0
Jinfen 1	61.5 ± 11.6	81.8 ± 0.6	85.4 ± 1.1	30.0 ± 12.7
Yuejiao 1	56.6 ± 17.8	78.6 ± 5.3	83. 9 ± 5.2	27.2 ± 13.3
Maiden	59.9 ± 1.3	73.5 ± 1.6	77.1 ± 1.6	17.1 ± 2.8
Meishi 1	59.6 ± 17.8	76.1 ± 6.0	82.1 ± 6.1	22.5 ± 12.3
Meishi 2	66.3 ± 10.5	81.1 ± 2.6	86.8 ± 2.4	20.5 ± 8.2
Poingo	58.2 ± 8.1	76.0 ± 2.6	81.7 ± 2.0	23.5 ± 7.3
Tigua	48.1 ± 8.6	78.2 ± 0.8	81.7 ± 0.7	33.6 ± 9.1
Yuejiao 2	48.4 ± 9.0	78.8 ± 3.8	79.7 ± 7.8	31.4 ± 8.7
Ingagara	53.5 ± 4.3	80.5 ± 5.0	84.6 ± 4.5	31.1 ± 2.2
Mpotogoma	64.1 ± 14.3	72.2 ± 13.2	85.6 ± 3.5	21.4 ± 10.9
Kazirakwe	68.6 ± 2.6	76.1 ± 0.9	80.5 ± 0.8	11.9 ± 3.1
Zhongjiao 8	49.9 ± 1.7	75.6 ± 4.7	81.9 ± 5.1	32.1 ± 6.2
GN 60A	56.7 ± 7.5	82.4 ± 4.4	87.0 ± 3.3	30.4 ± 4.2
Hongjiao 1	58.5 ± 9.9	73.6 ± 1.6	79.0 ± 1.9	20.5 ± 8.3

Data expressed as the mean and standard deviation. There is a non-significant difference among means in each column. T_o_: onset temperature; T_p_: peak temperature; T_c_: conclusion temperature; ΔT: gelatinization temperature range.

**Table 5:** Pasting property parameters of BRS from 16 banana cultivars.

Sample	A (°C)	B (BU)	C (CU)	D (DU)	E (BU)	F (BU)	B-D (BU)	E-D (BU)
Saba	78.1	239	237	211	261	253	28	50
Guangfen 1	77.5	275	275	250	326	331	25	76
Jinfen 1	77.9	261	253	247	311	315	14	64
Yuejiao 1	79.2	273	270	217	281	285	56	64
Maiden	75.8	62	60	57	68	71	5	11
Meishij 1	78.9	250	244	183	235	236	67	52
Meishij 2	79.4	264	263	176	224	236	88	48
Poingo	76.7	184	140	175	214	211	9	39
Tigua	74.2	328	315	267	360	358	61	93
Yuejiao 2	75.7	289	284	258	318	318	31	60
Ingagara	79.2	140	127	130	160	160	10	30
Mpotogoma	77.5	222	215	199	245	256	23	46
Kazirakwe	73.7	294	289	251	302	305	43	51
Zhongjiao 8	78.8	131	118	123	159	161	8	36
GN 60A	79.1	251	226	231	292	299	20	61
Hongjiao 1	72.7	182	182	156	201	202	26	45

Data expressed as the mean. A, initial gelatinization temperature; B, peak viscosity (BU); C, viscosity at 95 °C (BU); D, viscosity after 5 min at 95 °C (BU); E, viscosity at 50 °C (BU); F, viscosity after 5 min at 50 °C (BU); B-D: breakdown viscosity; E-D: setback viscosity.

**Table 6:** Results of cluster analysis of BRS characteristics from 16 banana cultivars.

Style	Name	Genotype
1	Saba, Mpotogoma, Kazirakwe	ABB, AAA, AAA
2	Maiden	AAB
3	Meishi 1, Poinga, Hongjiao 1, Ingagara, Zhongjiao 8	AAB, AAA, AAA, AAA, AAA
4	Guangfen 1, Yuejiao 1, GN 60A, Yuejiao 2, Jinfen 1, Meishi 2, Tigua	ABB, AAB, AAA, AAA, ABB, AAB, AAB

## Data Availability

The original contributions presented in the study are included in the article, further inquiries can be directed to the corresponding authors.
